# Wag the Dog: A Digital Literacies Narrative

**DOI:** 10.1177/23813377211027556

**Published:** 2021-07-21

**Authors:** Elizabeth (Betsy) A. Baker

**Affiliations:** 1 University of Missouri Columbia, MO, USA

**Keywords:** digital literacies, new literacies, literacy, technology, reading, writing, sociocultural theory, qualitative, grounded theory

## Abstract

In the spring of 2020, schools across the country and world closed. COVID-19 reached pandemic proportions. Were schools prepared? Was there a research base available to help schools prepare students for reading and writing digital texts? The ability to read, analyze, compose, and communicate with digital texts requires digital literacies. However, the rapid-fire development of information and communication technologies (ICTs) makes the identification of digital literacies and the development of curriculum and instruction a moving target. In her Literacy Research Association Presidential Address, Dr. Betsy Baker asserts that digital literacies are no longer an entity separate from reading and writing instruction, they are no longer a technology issue, students live in a digital world, and digital literacies are not optional. Digital literacies have become the literacies of our culture. Baker synthesizes over 25 years of research to propose that digital literacies are persistently public, semiotic, product-oriented, and transitory. Researchers, educational leaders, and teachers can leverage these characteristics as footholds to identify ever-changing digital literacies, design curricula, and provide instruction so that all students can be autonomous as they seek to thrive in a digital world. Dr. Baker’s Presidential Address is available online (see https://youtu.be/Avzup21ZnA4).

In 2005, Don Leu gave what I considered a riveting Literacy Research Association (LRA) Presidential Address (see Leu, 2006). He challenged the LRA community to consider two questions:

1. Why do schools *not* prepare students for the new literacies of the internet, especially in the United States and especially in economically challenged school districts?
2. Why do literacy researchers *not* focus their attention on the new literacies of the internet, helping schools to prepare students for their literacy futures? (p. 1)

Fifteen years have come and gone, and I wonder if these questions persist.

In the spring of 2020, schools across the country and world closed. COVID-19 reached pandemic proportions. Some schools provided worksheet packets for students to complete, some provided video conferencing between teachers and students, and some moved components of their curriculum to virtual learning. During the fall of 2020, we saw more of the same. The digital divide became more obvious as students who lacked opportunities to secure worksheet packets, join video conferences, or go online were declined access. Little is known about whether those who had online access also had the requisite digital literacies to engage in meaningful learning. I wondered whether schools were prepared and whether they had access to research that would support their efforts to prepare students for reading and writing digital texts. I speculated that digital literacies were foundational, even prerequisites, to online learning. I wondered if literacy researchers could provide answers to the daunting challenges encountered when schools were thrust online.

In an attempt to ascertain whether digital literacies have entered the public consciousness, I did what I think many of us would do, I did a Google search for the keywords, digital literacies. I found 3 million matches. I thought this was pretty good, but I was unsure what it told me. So, I refined my search by using Google Scholar. I limited my search parameters to the years since Leu’s Presidential Address, 2005–2020, and set the source parameters to journals. According to Google Scholar, in the past 15 years, 577,000 journal articles have been published. Next, I limited the search to “digital literacies” and found that .5% of these journal articles included the term, “digital literacies.” To be honest, I am not sure what this tells me either. I am not sure if the literacy research community is getting the job done or not. I do know that when faced with a pandemic, Leu’s questions resounded in my ears. I wondered: Can literacy researchers do more to support schools and prepare all students to be proficient readers and writers of digital texts?

I wonder if one roadblock is the perception that students know how to use digital forms of information and communication technologies (ICTs) better than the adults in the room. I often hear and repeat the following sentiment, “If you don’t know how to use [insert technology], ask a child or a teenager.” Evidence suggests, however, that students are proficient with the few technologies they and their friends use—but may lack a full spectrum of other capabilities (e.g., Auxier & Anderson, 2021; Vogels & Anderson, 2019). We cannot assume that they acquire or develop a robust range of digital literacies. To ignore a full complement of digital literacies in literacy research and schools is comparable to assuming children will develop alphabetic literacies and have no need for reading or writing instruction.

A strategy for making sense of a phenomena is to identify its basic characteristics. If you want to make sense of your favorite flower, or the value of exercise, or maybe a persistent threat like coronavirus, you need to identify the phenomena’s defining characteristics. Throughout my career, I have sought to identify some of the defining characteristics of literacy in our digital world. The characteristics that I have identified are shaped by sociocultural tenets (Cole, 1996; Heath, 1983; Moll, 1990; Rogoff, 1995) that have emerged primarily while I conducted naturalistic studies (Hammersley & Atkinson, 2007; Heath & Street, 2008; Lincoln & Guba, 1985). It is my hope that a brief exploration of these characteristics will provide footholds that we can use to support schools and students as we continue to live in an ever-increasing digital world.

I describe four characteristics of digital literacies around which literacy researchers and educational decision makers might coalesce to support students and schools. None of these are surprising or earthshaking until we peel back some layers, dig deeper, and scrutinize the implications for literacy research, learning, and pedagogy. The purpose of this article is to consider the nature of literacy in our digital world as well as brainstorm how literacy researchers might join efforts to support students and schools.

Based on theories of anchored cognition whereby learning is garnered through a shared experience (Baker, 2009; Baker & Wedman, 2007), I recommend that you watch the trailer for the movie, *Wag the Dog* (Levinson, 1997; see https://youtu.be/steA_PZPkc8). This movie was an adaptation of Beinhart’s satirical novel, *American Hero* (1993). The basic plot involves the American President caught in a scandal just before an election, so a spin doctor creates a fake war to distract the American press. At the conclusion of this article, I reference this trailer to illustrate some basic characteristics of literacy in our digital world.

## Thanks Are Due

Before I explain the illustrations of digital literacies that I derive from *Wag the Dog*, many thanks are in order. I am deeply indebted to my parents, Maude and Walt Baker, who fostered a love of learning, curiosity, and advocacy. My elementary school colleagues who supported me as I strove to promote autonomy for all learners. My graduate school mentors, Tom Cloer, Vicki Risko, Debbie Rowe, and John Bransford among others, who invited me to become a member of supportive and invigorating graduate communities and quickly became not only mentors but also friends. My advisor Chuck Kinzer. I warned Chuck that once he was my advisor, he would always be my advisor. He mentored me into LRA and the field. During invigorating conversations about my research dreams and aspirations, I can still see Chuck lean back in his brown Naugahyde swivel chair, smile from ear to ear and ask, so what? Why should anyone care about the research you are conducting? This has been a useful heuristic to live by. Thanks Chuck! I wouldn’t be here without you.

I thank Don Leu, Linda Labbo, David Reinking, Mike McKenna, and Donna Alvermann, to mention a few, for forging the pathways of new literacies and digital literacies scholarship. You are preeminent among the scholarly giants on whose shoulders I stand. I thank the many editors and reviewers through the years of *LR: TMP, JLR, RRQ,* and other publications who patiently and gracefully mentored me through the publishing process. Knowingly and unknowingly, many LRA members have mentored and continue to mentor me. I read your articles, listen to your podcasts, attend your conference sessions, work with you on committees, and collaborate with you to conduct and disseminate research. Your work invigorates and inspires me! LRA has been my academic home for over 25 years. You are my research community. It is a privilege to be part of LRA.

## Threads Through Time

If reading and writing are social acts and cultural norms are situation bound (Cole, 1996; Heath, 1983; Moll, 1990; Rogoff, 1995), I wondered: What is literacy in our technology imbued culture? How could I be a literacy researcher and teacher educator if I did not know what constitutes the essence of literacy in our increasingly digital world? Using grounded theory (Birks & Mills, 2015; Charmaz, 2006), I revisit four characteristics of literacy that emerged during my dissertation and pull these threads through time. It is my hope to grapple with the tectonic shifts we have encountered during the digital revolution and expose implications for reading and writing in our digital world.

### The Public Nature of Literacy

Using ethnographic research methods in a fourth-grade classroom where students were outnumbered by computers, there was evidence that a basic characteristic of reading and writing was that it was public. During interviews, students made such comments to me as:

Betsy: Do you usually look at each other’s screens?

Simon: From my seat, I can see Bert’s, Jacob’s, and Jesse’s computers.

Lisa: Yes, so I can see what [my classmates] wrote.

Jackie: [My classmates] are curious about what [my story] was about. People come by and look at [my computer screen].

Ms. Jones: I think there are times that they do learn from each other. Carolyn…observed other people. Well, she had to do that for a while. And then when somebody actually taught her the steps to go through [to use an app], you saw her comfort level…she was tickled, so tickled. (Baker, 1995, p. 107)

The teacher, Ms. Jones, explained,

I definitely think that the writing is public [in this classroom]. The disadvantage of [compositions] being up on the [computer] screen, I think sometimes children in their journal writing [are] not as open…In fact, I did journal writing with second graders last year [without computers]. They wrote in their notebooks every day. And so, they knew that the notebook was theirs and they gave me permission to read it. If I got permission to read it, I read it. Otherwise I did not read their journals. These children [using computers], when they do journal writing, it is more for a reader—somebody else to read it. It is putting our thoughts down on paper, expressing ourselves, but you know…you are going to have an audience…I definitely think that they walk around and read each other’s screens. (Baker, 1995, pp. 114–115)

In this interview, Ms. Jones highlighted the notion that not only was literacy public but that this publicness altered what students wrote. The second graders who wrote on paper had their privacy protected. The fourth graders who composed on computers could see one another’s screens from where they sat. On one hand, students learned from each other when walking by and reading each other’s screens. On the other hand, Ms. Jones believed that they filtered what they wrote because they had both solicited and unsolicited audience members.

#### Digital culture since the 1990s

Fast forward 20 years: Working with a team of researchers, we parsed the literature to consider whether the public nature of literacy in our digital culture remained a viable construct (see Baker et al., 2015). To pull this thread through time, let me first consider where we were as a digital culture in the 1990s (see Roser et al., 2015). The first web page was released in 1991. Within 4 years, Mosaic, Netscape, eBay, and Yahoo were online. Internet Explorer and Amazon were released in 1995. Google came online in 1997, Skype in 2003, Facebook in 2004, and YouTube in 2005. In 2006, Twitter was launched. In 2007, the iPhone came out. By 2010, we had WhatsApp, Uber, Bitcoin, and Instagram.

User data increase followed suit. It is estimated that in 1990, half of 1% of the world population was online (Roser et al., 2015). By 2016, approximately 3.4 billion or 46% of the world’s population had used the internet in the previous 3 months. In 2016, the U.S. ranking for percent of the population that had used the internet within 3 months was tied with Lebanon and the Czech Republic as 33rd in the world with 76% of the population online. By 2021, 93% of U.S. adults reported using the internet (Perrin & Atske, 2021). It is estimated that between 2010 and 2016, on average, each day 640,000 people went online for the first time which equated to 27,000 new users each hour (Roser et al., 2015). As of 2020, I am going to guess that on any given day, it is likely that you will pick up a digital device to communicate or find information.

#### Twitter and TV

One of my favorite examples of the public nature of digital literacies is work done by MIT Professor and Executive Director of the MIT Media Lab, Dr. Deb Roy. From 2013 to 2017, Roy served as Twitter’s Chief Media Scientist. Roy and his team examined the transactional relationship between TV viewing and Twitter participation. In this case, Roy follows tweets that occurred during the British TV show, *The X Factor*. Roy (2014) uses dataviz to trace the life of one tweet. For example, Clare Hardy, tweets,

Sharon Osbourne is just the best. EVER.

I’m laughing uncontrollably. #XFactor.

**Figure 1.**
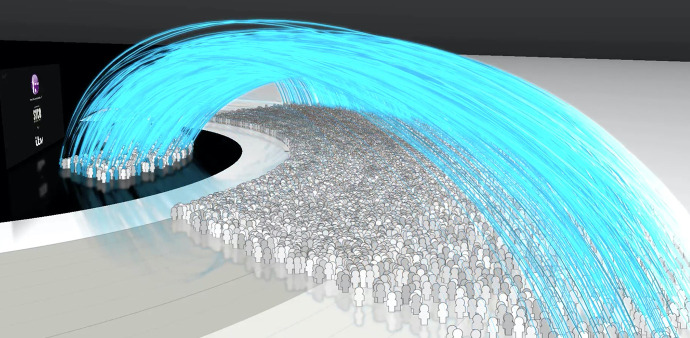
Tsunami of shared experiences. *Note*. TV viewing audience shown in the dark inner circle; posting tweets shown in arching lines from center to periphery; Twitter audience members shown in gray and white bands. *Source:* Roy (2014), used with permission.

Clare Hardy posted her first tweet at about 8:30 p.m. Within a few seconds, someone reads Clare’s post. Over the next couple of hours, Clare Hardy’s tweet is picked up by others and extends even beyond the broadcast viewing time. So far, the public nature of literacy is evident by noting Clare’s ability to post to Twitter and her post is read by several people. But I want to push the public nature of digital literacies a bit further. Roy takes a random sample of 1% of the TV viewing audience and traces their Tweets. Roy describes a virtual tsunami of shared experiences between the TV viewing audience and Twitter users (see Figure 1).

Let me review. Clare, while watching *The X-Factor*, posted a tweet and that tweet had an audience. Thus, her digital composition was public. When 1% of the viewing audience was followed, Roy traced a virtual tsunami of public communication between TV viewers and Twitter users. Let me push the public nature of digital literacies even further.

**Figure 2.**
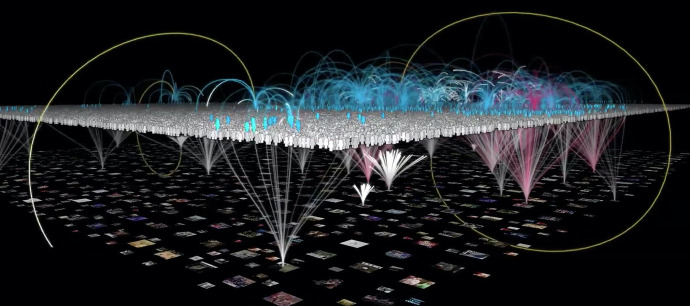
Percolation of communication. *Note*. Twitter users are represented in the white layer, TV broadcasting is represented in the bottom layer, with a percolation between layers represented by arched lines. *Source:* Roy (2014), used with permission.

Roy (2014) describes Andrew Kitzenberg’s experience: One evening, while watching the local news, Andrew looked out his window and realized he was witnessing the manhunt that ensued after the Boston Marathon bombing. Andrew took to Twitter to describe what he saw out his window. His Tweets were picked up and he became part of live television. Roy describes how the TV audience, became Twitter authors, who became TV participants. He describes the author/audience transaction between TV and Twitter as percolation of communication (see Figure 2).

Not only did Clare Hardy have an audience, not only did 1% of the *X-Factor* tweets become a tsunami, but Andrew Kitzenberg became part of the TV news. These examples are derived from only two media streams: television and Twitter. As we all know, these are but two of a plethora of digital communication platforms. In further analysis of the public nature of digital literacies, colleagues and I examined the shifting role of audience and audience awareness and noted how in a digital world the role of author and audience is blurred—which raises all sorts of intriguing research questions that are rife with pedagogical implications (see Baker et al., 2000).

#### Viral videos and social media challenges

In addition, the public nature of digital literacies is evident when we refer to online posts, hashtags, and videos that have gone viral. Who can forget the ALS Ice Bucket Challenge? Such examples are now common: the Planking Challenge, the Running Man Challenge, the Mannequin Challenge. On a recent Milwaukee news broadcast (TMJ4 News, 2020), there was mention of someone who wanted to support restaurant workers who are facing difficult times due to COVID. She created a Venmo Challenge to give almost $5,000 to area servers and delivery drivers.

#### Grounded theory: Public

Given the persistence of the public nature of literacy over the last 25 years, I am going to do something that is risky. I am going to attempt to predict the future. I conjecture a grounded theory:

The nature of literacy in our digital culture will continue and even become increasingly public.

While such predictions are in danger of current and future ridicule, if there is a glimmer of insight, such predictions may help us to prepare for the future.

#### Collective and artificial intelligence

Consider how the public nature of literacy aligns with advances being made in collective and artificial intelligence. In 2015, my research team wrote,

While collective intelligence is as old as society and exhibited by most forms of life (Malone & Bernstein, 2015), due to the public nature of digital communication, we are on the precipice of something previously unattainable. (Baker et al., 2015, p. 99)

Consider Wikipedia where editorship belongs to the collective—or more recently, to the approved collective. Consider GoFundMe, KickStarter, Indiegogo, and DonorsChoose where innovators and funders can find each other. There are Massively Multiplayer Online Games (MMOGs) and Massively Multiplayer Online Role Playing Games (MMORPGs) that are inherently public. From 2008 to 2015, the International Genome Project solicited voluntary DNA uploads that were used to examine the age-old quandary regarding the role of nature versus nurture in disease. There are online ratings that shoppers, moviegoers, and university students can consult. There are ride-share apps such as Uber and Lyft that help you find strangers who are willing to give you a ride.

It is said that during the era of Web 1.0, readers read the Internet. During the years of Web 2.0, readers and authors collaborated. More recently, in the era of Web 3.0, given browser cookies and such, the internet reads the readers (Baker et al., 2015; Markoff, 2006). All iterations require proficiencies with the publicness of digital literacies and have implications for literacy researchers and schools.

There are affordances and challenges to the public nature of digital literacies. There is online bullying and disinformation. In 2014, Pew reported that 73% of adult internet users have witnessed online bullying while 40% have experienced it (Duggan). Anyone living in a country that allows open access to the internet can post to social media, give online ratings, as well as find and create collective intelligence. On the other hand, search engine algorithms arguably monopolize what we find online. At the click of a button, your site, your posts, your collaborations, your collections can be made impossible to find or even removed. Greenwald (2014) points out that simultaneously, online communication and information are democratized and monopolized by stating, “We are nearing the point where an idea banished by Twitter, Facebook, and Google all but vanishes from public discourse entirely” (para. 12). Such possibilities require in-depth literacy research and adroit pedagogy. Given evidence that literacy is and continues to be inherently public, there is a clarion call for ongoing research to examine corresponding implications to prepare all learners to proficiently read and write in our digital world.

### The Semiotic Nature of Literacy

At the turn of the millennium, reading research transitioned from focusing on reading alphabetic texts to embracing the symbiotic relationship between reading as well as writing alphabetic texts. To demonstrate this shift, the National Reading Conference changed its name to the Literacy Research Association. The *Journal of Reading Behavior* became the *Journal of Literacy Research*. Definitions of literacy continued to expand. In 1994, Rowe leveraged semiotics to position preschoolers’ scribblings as meaning-filled written expressions thus laying the groundwork for literacy scholars to embrace multimodal texts (see also Clark, 1990; Leland & Harste, 1994; Short & Kauffman, 2000). In the ensuing years, literacy scholars similarly drew on semiotics to consider the reading and writing that occur when users encounter multimodal digital texts (see Baker, 2000; Gillen, 2014; Kontovourki & Siegel, 2021; Kress, 2010; Serafini, 2014; Siefkes, 2015). In 1995, I observed Sally as she researched Alaska.

[To learn about Alaska,] Sally…read her social studies textbook…which incorporated alphabetic text, illustrations, and photographs. Sally used this information to develop the parameters of her investigation and acquire key words…[Online] she found video clips of Alaskan lifestyles, interviews with Alaskans, and views of terrain and weather conditions including sound effects of harsh winds and frozen lakes cracking during spring thaws…Finally, Sally searched the internet and found additional sources that incorporated various sign systems. (Baker, 1995, p. 170)

This excerpt describes how Sally relied on multiple sign systems to understand life in Alaska.

These sign systems were not merely different representations of the same information—they were interdependent representations. Specifically, the interviews with Alaskans revealed to Sally how their language was different [from her own]. The video of the terrain exposed Sally to how rocky and icy the mountains were. The howl of the wind and deep moans of the cracking ice demonstrated how Alaska could be bitterly cold and different from her climate. These sign systems were interdependent and presented Alaska to Sally in ways that alphabetic text, alone, could not. (p. 170)

While reading alphabetic texts can be viewed as a psycholinguistic guessing game (Goodman, 1967), digital literacies might be viewed as a semiotic guessing game (Baker, 2001), where we simultaneously sample alphabetic, linguistic, visual, auditory, gestural, and spatial cues (New London Group, 1996).

In 1999, the Biography Channel did a countdown of the most influential people of the second millennium (1000–1999 AD) (see Zad, 1999). Among others, they featured biographies of Susan B. Anthony, Peter the Great, da Vinci, Mozart, Darwin, and Gandhi. They concluded this series by identifying Gutenberg as the most influential person of the second millennium. They argued that the ability to mass produce and distribute the written word transformed the world. Some say that we are experiencing a Gutenbergian Revolution (Patton Howell, 1990; Wheeler, 2019). Digital texts—with the ability to encode and share alphabetic, linguistic, visual, auditory, gestural, and spatial expressions beg the question: What is literacy in our digital culture? Are literacy teachers now expected to help students develop not only alphabetic reading and writing skills but also help students proficiently communicate with digital texts that incorporate visual, auditory, spatial, and embodied sign systems? Should literacy teachers be teaching art, music, theater, and video production? Should there be a hierarchy of sign systems where we target alphabetic texts in the early grades and other sign systems in later grades? Should all sign systems, and the orchestration of all sign systems, be part of the literacy curricula K–12?

I am intrigued by varied notions of time and space as they relate to literacy (e.g., Baker, 2010; Bakhtin, 1981; Compton-Lilly & Halverson, 2014; Leander & McKim, 2003). Time travel captivates the imagination. From H.G. Wells’ (1895) classic, *The Time Machine*, to the successful franchise, *Back to the Future*, our culture has been riveted by time travel. For decades, even centuries, humankind has searched for a time machine. I have seen proclamations that the time machine has been invented; it is called reading and writing (e.g., Dickinson, n.d.). Given a time/space perspective, it can be argued that alphabetic text defies time and space. We can read the words of Socrates, Tertullian, and Confucius without living in ancient Greece, Africa, or China. When children proficiently read and write alphabetic text, they can travel through time and space.

Semiotic sign systems are not new. Cave drawings, petroglyphs, and pictograms indicate that humanity has communicated with multiple sign systems for millennia. What is new is that multimodal texts now travel at lightning speed through space. I can text, email, and post to social media. Wherever you are in the world, you can receive my message within seconds.

I teach an online graduate course in which we examine varied ICTs and attempt to be metacognitive about the literacies we use. Fourteen years ago, I was able to move along a continuum of verbocentric to gestural texts. It is now a challenge to separate sign systems from ICTs. For example, we used to examine the literacies that were needed to read and write text messages, email, search engines, Twitter, and back channels. At the time, these were commonly alphabetically based ICTs. We progressed through multimodal ICTs such as online catalogs, news websites, and Facebook, and proceeded to visually based ICTs such as Instagram and YouTube. Then we discussed the digital literacies of augmented realities as well as virtual realities where users interact as avatars.

A few years ago, I had to restructure the course because this continuum grew opaque. Text messages, emails, and Twitter contained emojis, giphys, photos, and videos. The days of reading as a psycholinguistic guessing game while making sense of alphabetic text now reside within a broader multimodal world. Research is needed to examine the semiotic guessing game.

How do the reading and writing processes of psycholinguistic and semiotic guessing games compare? Researchers might explore the relationship between sign systems as well as how readers and writers orchestrate sign systems.

**Figure 3.**
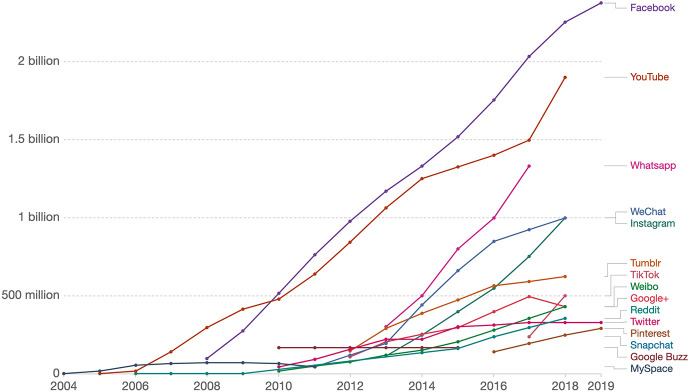
Number of people using social media, 2004–2019. *Note*. Users are defined as those who have logged in during the past 30 days. *Source:* Ortiz-Ospina (2019), used with permission.

From 2004 to 2019, the number of people using social media went from zero to nearly 3 billion people (Ortiz-Ospina, 2019; see Figure 3). Starting from the bottom, to mention a few: Snapchat, Pinterest, Twitter, TikTok, Instagram, YouTube, and Facebook. Consider for a moment, what is the role of alphabetic text in these platforms? As a side note, I wonder whether emojis should be part of vocabulary instruction.

In 2013, 7 years ago, the United Nations reported that worldwide, more people owned cell phones than had access to functioning toilets (Wang). Specifically, at that time, it was estimated that 6 of the approximately 7 billion people on earth had cell phones while only 4.5 billion had functioning toilets. In 2019, Pew reported that 96% of Americans own a cell phone and 81% of these are smartphones which support multimodal communication through texts, emails, social media, and the like.

I have described the ability of digital texts to travel through space at the speed of light. We know less about the duration of digital texts and their ability to travel through time. I have books on my shelves that are over 100 years old. I hope my Facebook posts are not available in 100 years.

Linguists argue that babies persist through the challenges of learning oral language because of the innate need to communicate. Likewise, because written artifacts can travel through time and space, authors persist with the craft of written expression. Now that multimodal texts travel through time and space we are witnessing the explosion of digital information and communication. I wonder what role time and space have in the ontology of literacy. Might reading and writing exist to afford travel through time and space? If this is the case, given a continued exponential increase in ICTs, it seems plausible that digital literacies and those who develop digital literacy proficiencies are the ones who are poised to ride the time machine.

It is, therefore, imperative that we help students proficiently produce and make sense of all forms of texts. In 2006, Leu stated, “the internet is a reading and literacy issue, not a technology issue” (p. 6). May I be so bold as to update this statement by asserting,

Text messages, emojis, giphys, animations, email, internet searches, internet browsing, hyperlinks, discussion boards, wikis, Google Docs, podcasts, Twitter, Facebook, Snapchat, Instagram, LinkedIn, YouTube, TikTok, livestreams, virtual field trips, MMOGs, MMORPGs, augmented realities, virtual realities, virtual currencies, collective intelligence, artificial intelligence and the like are not merely technological progressions but literacy progressions.

In 1995, I concluded that Sally’s multimodal investigation of Alaska was an example of the semiotic nature of literacy in a digital classroom. In the ensuing years, the semiotic nature of literacy has undergone a Gutenbergian revolution. While the specific apps and technologies will come and go, based on the persistence of the semiotic nature of literacy in our predigital and digital culture, I conjecture a second grounded theory:

The nature of literacy in our digital culture will continue and even become increasingly semiotic.

How can we prepare preservice teachers, support in-service teachers, and help educational leaders navigate the semiotic literacy landscape? What research is needed to help students travel through time and space in our digital world? How can we, a community of literacy researchers, provide the requisite research, teacher preparation, and educational support?

### The Product Nature of Literacy

Donna: I’ve written a whole story!

Maggie: I like doing this! I wish I could write every day!

Alyson: This is cool!

Eric: I am gonna need that Dragon because there is a bunch of hard words.

These are quotations from a current research project where first graders use Siri, Dragon, Alexa, Hey Google, and other speech recognition (SR) apps to compose (see Baker, 2017, 2019; Baker & Bradley, 2019; Baker & Goodwin, 2019). Typically, we think of these apps as online assistants. While driving, we can say, “Hey Siri/Alexa: Call Tom, will it rain today, how do I get to the closest McDonald’s?” But SR can also take dictation. Children can talk and watch their robust cultural vernacular magically appear on the screen. I like to say, they can *Talk to Read*© (Baker, 2014).

In 1995, I found that the nature of literacy in a technology-rich classroom was public, semiotic, and the third thread I want to pull through time is that literacy was product-oriented. When I asked the fourth graders in Ms. Jones’ classroom whether they preferred to compose with a computer or paper and pencil, they made such comments as,

Sally: It is easier and neater to read [typed stories].

Melissa: People cannot read cursive as well.

Sally: It would take longer [to publish with paper and pencil] because I worry about handwriting.

Trevor: [Using technology] is easier and more fun than pencil and paper. For example, you can type faster than write [with paper and pencil]. (Baker, 1995, pp. 155–156)

These students explained that computers mitigated challenges they encountered when writing and reading handwritten texts. When asked about composing multimodal texts, students made such comments as,

Carolyn: I like it better [with imported graphics and video] because you do not have to draw it by hand. I can’t draw that good. I can’t draw anything.

Betsy: What if you had to do a picture without [imported graphics]?

Josie: I could not do it.

Sally: It is easier to [illustrate] on computer.

Simon: [With computers,] you can copy it and move it. [Student showed me how to cut and paste clip art.]

Randy: Drawing with a computer is easier. (Baker, 1995, pp. 156–157)

These excerpts reiterate that literacy in this classroom was public, semiotic, and product-oriented. This could be a vestige of the classroom norms because the curriculum was inquiry-based with projects that culminated in presentations. In other words, the students were expected to share what they learned during their inquiries. To do so, they considered what products would best communicate their learning. Would a PowerPoint with bulleted alphabetic text be a good product or should they create an animation or show video clips?

Here is another research topic: What digital literacies are needed to make a match between digital products and the message you want to communicate? In Ms. Jones’ classroom, they had to consider the affordances of digital products. When, why, and how can students choose one digital product over another? When, why, and how can students make PowerPoints, websites, podcasts, blogs, videos, or social media posts? I propose that genre studies may open a doorway into digital literacies pedagogies.

You are beginning to see that it is difficult for me to claim that literacy in this classroom was only public or only semiotic or only product-oriented. Simultaneously, reading and writing were all of these things. To describe literacy as public and semiotic without considering the product orientation would be to ignore an important facet of literacy.

In my current work with SR and *Talk to Read*©, the first graders learned that they could record oral renderings of their stories. When I invited them to share their recordings with classmates they were commonly aghast. Their audio products were not for classmates or teachers but for themselves. It may be more accurate to say that they wanted to be their own audience. They wanted to create a product that they could revisit. They wanted to listen to their oral recordings of their readings and relish their increased reading fluencies. The product gave them this ability. The product traversed time and space. While the public nature of literacy provides an audience, the product nature allows authors to not only share but also revisit their work (see Salomon, 1993). As a potter needs to fashion clay, the authors in these various studies needed to fashion products. Technology provided product-oriented affordances that paper and pencil lacked.

My work in 1995 indicated that students composed public and multimodal texts because they could make products. In my current work with first graders using SR to compose, the technology mitigates challenges to encoding their personally meaningful texts. The first graders are autonomous, self-directed, as they dictate emails to their parents, their own versions of familiar stories, songs, and poems as well as journal entries and creative stories. They cherish the products that technology makes possible. The third characteristic of literacy in a technology-rich fourth-grade classroom was that it is product-oriented. Based on the ensuing decades and continued analyses, I propose a third grounded theory:

The nature of literacy in our digital culture will continue and even become increasingly product-oriented.

Whether you take photos on your smartphone, post to social media, or craft a video for YouTube or TikTok, all are opportunities to create products. Because products traverse time and space, it is worth noting that the ethereal, what previously lacked substance, now has substance. Conversations, research presentations, even walks through the park can be captured and shared.

In his popular 2011 TED Talk entitled *The Birth of a Word*, Roy described his longitudinal efforts to examine language acquisition. Using their first child, a son, as his subject, Roy and his wife installed surveillance cameras throughout their house. For 3 years, they video-recorded 8–10 hr/day and collected a quarter-million hours of multi-track audio and video. Roy describes this data set as, “The largest home video collection that was ever made” (1:52). He also describes the methodological challenges encountered with making sense of 90,000 hours of video.

I am describing Roy’s (2011) work to illustrate how he captured the ethereal to create a product that traversed time and space and therefore leveraged affordances that allowed researchers to examine what he calls, the birth of a word, that would previously have evaporated into thin air. Roy crunches 6 months of data into 40 seconds. His son transforms his oral language from “gaga” to the socially understood word, “water” (see Baker, 2020, 55:10-55:56). I highlight this audio sample to demonstrate how the ethereal, the oral word in this case, can become a product and travel through time and space for us to listen to today. While this is wonderful, the fact that oral language can be recorded certainly is not new. I want to use this recording of oral language to dig a bit deeper.

**Figure 4.**
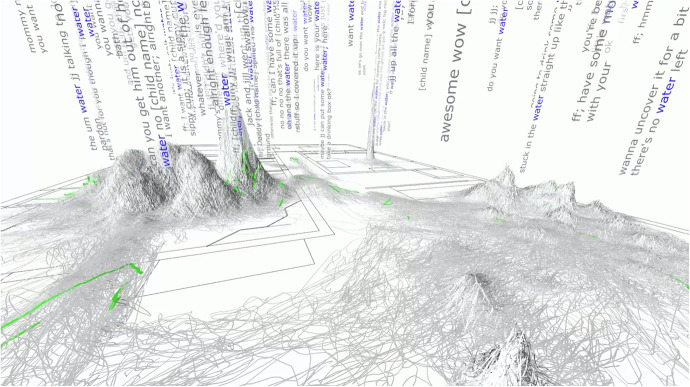
Wordscapes: Tracing word usage across time and space. *Source:* Roy (2011), used with permission.

To examine the role of social interactions and context on language development, Roy (2011) traced the location and interactions for each time his son was exposed to the word, “water.” Digital technologies are used to trace his son’s movements across time. In the video, his son leaves a red trace (see Baker, 2020, 56:38-57:37). His nanny enters the room. Her movements are traced in green. In the video, Roy and his team tag each instance of the word, water, which is traced through the floorplan of their home. Each tag is then followed across time and space to become what Roy calls, wordscapes (see Figure 4). Herein, the ethereal is not only captured as an audio file but is embellished with optics that allow researchers to examine the origins of oral language acquisition. Figure 4 illustrates that the word, water, while used throughout the home, was most commonly used in the kitchen.

Before the ubiquity of internet-connected mobile devices, conversations, actions, and settings were fleeting—they lacked substance, they were ethereal. In our digital world, the ethereal can become a product that similar to the written word can now travel time and space. I see research opportunities: What are the implications of the product nature of literacy? How can we prepare preservice teachers, support in-service teachers, and help educational leaders navigate the product-oriented landscape of reading and writing in our digital world so that all students can harness the potential of digital products?

### The Transitory Nature of Literacy

When I entered the classroom in January, Simon showed me the animation [he created] of Patrick Henry’s speech in the House of Burgesses. Simon had created this animation several weeks before I started data collection. When showing this animation to me, he noticed that the setting could use some revision (i.e., include a reference to the House of Burgesses, the date and candlelight). Such revisions highlight that the compositions in this classroom were not static—they were transitory. Throughout the year they were revised. (Baker, 2011, p. 3)

Digital literacies are not only public, semiotic, and product-oriented but also what I call, transitory. In other words, in this fourth-grade classroom, compositions were always in flux. This seems to contradict the product nature of literacy. However, it is actually an extension.

In the old days, using an actual typewriter, I typed papers for my teachers and that was it. I turned the papers in and never updated them again. I read newspaper articles and apart from the occasional corrections published the following day, the articles remained stable. In Ms. Jones’ technology-based classroom, students revised their animations and other digital compositions long after they were presented to the class and graded by the teacher. When I asked, “would you rather write on a computer or with paper and pencil?” students made the following comments to me,

Josie: Computer because it is easier to erase, like if you mess something up then you can just delete it instead of erasing it or marking it out.

Lisa: Computer because all you have to do is move up there and change your mistakes.

Tom: If you draw with a marker you cannot erase, but [with] a computer you can.

Teri: The computer can change it; with markers you start over. (Baker, 1995, p. 147)

I hesitate to point out that it is easier to revise digital texts than texts written on paper with pencil and marker. We all know this, right? However, when the transitory nature of literacy is taken together with public, semiotic, and product-oriented characteristics, some interesting implications emerge.

Twenty years later, my research team examined whether this construct persisted (Baker et al., 2015). We considered Wikipedia. The basic notion is that a worldwide set of editors can generate a broader storehouse of human knowledge that is continually updated than any central organization such as *World Book*, *Funk and Wagnall*, or *Encyclopedia Britannica* could possibly generate. Of course, the primary feature of wikis is that approved contributors can edit the same document. The same is true of Google Docs. In other words, unlike nondigital products, digital products can be transitory. They may change. The website that I visit today may have been updated, altered, and have different content tomorrow.

**Figure 5.**
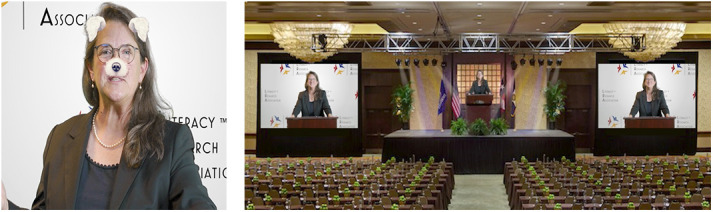
Transitory nature of digital texts. *Note*. To illustrate the transitory nature of literacy, during the 2020 Literacy Research Association Presidential Address, Baker used facial filters as well as virtual backgrounds of the intended venue, which was abandoned due to COVID-19 restrictions.

Of course, a range of digital literacies come into play when composing or making sense of transitory texts. Online sources may have been tampered with. Readers must understand how to ascertain what is authentic. The transitory nature of literacy has come to the forefront in our political world where naïve inaccuracies as well as malicious disinformation can be made to appear authentic. We all enjoy SnapChat, Instagram, and other app filters. While video conferencing or video recording, we can use virtual backgrounds. Our faces and backgrounds are transitory (see Figure 5).

The final characteristic of literacy in a technology-rich fourth-grade classroom was that it is transitory. Based on the ensuing decades and continued analyses, I propose a fourth and final grounded theory:

The nature of literacy in our digital culture will continue and even become increasingly transitory.

Whether you are creating an animation of Patrick Henry’s famous speech, reading or writing a Wikipedia article, perusing or posting to social media or a video-sharing platform, there are significant digital literacies involved in reading and writing transitory texts. Readers and writers of digital texts are autonomous or disenfranchised by their ability to navigate the transitory nature of digital texts.

## Conclusion

In conclusion, I can hear Chuck, as well as some of you, ask: So what? Why should anyone care? Let me attempt to respond. If the nature of literacy in our digital culture continues and even becomes increasingly public, semiotic, product-oriented, and transitory (PSPT), then we might have some footholds to formulate research and support schools. For example, in our digital world,

- What do students need to know about audience—solicited and unsolicited?
- What do they need to know about compositions that can go viral for good and bad reasons?
- What strategies can they use to remain safe while creating collaborative online communities that promote dialog and reject bullying?
- How can students become savvy readers who readily identify misinformation and disinformation?
- How can students leverage the written word, video, music, sound effects, voice-overs, illustrations, photographs, graphs, data vis, and more, to wax eloquent and convey their insights to a worldwide or singular audience?
- What do students need to join or even invent the collective that heretofore was nonexistent?
- What is needed for students to ride the time machine that is increasingly digital?
- How can they create digital products that traverse time and space in ways that were previously unattainable?

We cannot assume that because students live in a digital culture that they will develop the requisite digital literacies. We cannot wait until students enter middle school or high school to support digital literacies learning. I applaud the revisions made to the International Literacy Association (ILA) Standards (2018a) which emphasize the need to prepare preservice and in-service teachers as well as literacy specialists to understand and teach digital literacies. I underscore the ILA bi-annual survey of hot topics which in International Literacy Association 2018b found the #1 topic was digital literacies. While I do not claim that the PSPT characteristics I have described today are the only characteristics or footholds that might capture the essence of literacies in a digital world, I propose that those who harness the potential while mitigating the challenges of digital literacies are poised to thrive in our culture. We can sit on the sidelines or lead the way.

## Wag the Dog: A Digital Literacies Narrative

At the beginning of this article, I encouraged you to watch the trailer for the movie, *Wag the Dog* (https://youtu.be/steA_PZPkc8). Let me explain. As you recall, the spin doctor states that he needs “a theme, a song, some visuals—you know, a pageant” (0:48-0:52). The spin doctor harnesses the *public* nature of digital literacies to distract the press until the election. He creates a *semiotic product* complete with verbocentric dialog, auditory screams, and embodied as well as musical texts that are *transitory* when he inserts an Albanian village in the background and converts a bag of chips into an adorable kitten. The book became a movie which became a trailer that was posted online. To appear here, the product traversed time and space. To be clear, I am not advocating that we prepare students to be the best spin doctors they can be; rather, we prepare students to be proficient, even savvy, readers and writers, consumers and producers of digital texts.

Whether communicating by text messages, emojis, giphys, animations, email, internet searches, internet browsing, hyperlinks, discussion boards, wikis, Google Docs, podcasts, Twitter, Facebook, Snapchat, Instagram, LinkedIn, YouTube, TikTok, or livestreams, to mention just a few, we must provide the requisite research as well as advocate for the autonomy of all learners to thrive in our digital culture. As literacy researchers, there may be no greater honor than to support students’ abilities to orchestrate information as well as communicate.

When contemplating a title for this article, I vacillated between including and deleting the word, “digital.” I concluded that when I struck out the word, digital, it represented my point: digital literacies are no longer a separate entity. They are no longer a technology issue. Students live in a digital world. Digital literacies are not optional. Digital literacies have become the literacies of our culture.

I leave you with this: The questions resound. Can we do more? Can we do better? Can we support schools and prepare all students to be proficient readers and writers of digital texts?
